# Different modulation by dietary restriction of adipokine expression in white adipose tissue sites in the rat

**DOI:** 10.1186/1475-2840-8-42

**Published:** 2009-07-30

**Authors:** María del Mar Romero, José Antonio Fernández-López, Montserrat Esteve, Marià Alemany

**Affiliations:** 1 Department of Nutrition and Food Science, Faculty of Biology, University of Barcelona, Barcelona, Spain; 2 CIBER Obesity and Nutrition, Carlos III Institute of Health , Spain

## Abstract

**Background:**

White adipose tissue (WAT) is a disperse organ acting as energy storage depot and endocrine/paracrine controlling factor in the management of energy availability and inflammation. WAT sites response under energy-related stress is not uniform. In the present study we have analyzed how different WAT sites respond to limited food restriction as a way to better understand the role of WAT in the pathogenesis of the metabolic syndrome.

**Methods:**

Overweight male rats had their food intake reduced a 40% compared with free-feeding controls. On day ten, the rats were killed; circulating glucose, insulin, leptin, adiponectin, triacylglycerols and other parameters were measured. The main WAT sites were dissected: mesenteric, retroperitoneal, epididymal and subcutaneous inguinal, which were weighed and frozen. Later all subcutaneous WAT was also dissected and weighed. Samples were used for DNA (cellularity) analysis and mRNA extraction and semiquantitarive RT-PCR analysis of specific cytokine gene expressions.

**Results:**

There was a good correlation between serum leptin and cumulative WAT leptin gene mRNA, but not for adiponectin. Food restriction reduced WAT size, but not its DNA content (except for epididymal WAT). Most cytokines were correlated to WAT site weight, but not to DNA. There was WAT site specialization in the differential expression (and probably secretion) of adipokines: subcutaneous WAT showed the highest concentration for leptin, CD68 and MCP-1, mesenteric WAT for TNFα (and both tissues for the interleukins 1β and 6); resistin was highly expressed in subcutaneous and retroperitoneal WAT.

**Conclusion:**

Food restriction induced different patterns for mesenteric and the other WAT sites, which may be directly related to both the response to intestine-derived energy availability, and an inflammatory-related response. However, retroperitoneal WAT, and to a lower extent, subcutaneous and epididymal, reacted decreasing the expression of inflammatory markers and the signaling of decreased energy availability in their stores. The varying cytokine expression patterns highlight the fact that WAT sites show different inflammatory and signaling responses to energy availability; they are too much different to simply extend to the whole-body WAT the findings of one or even a couple of sites.

## Background

White adipose tissue (WAT) is currently considered a fairly dynamic tissue, which behaves largely as a disperse "organ" [1] with critical energy-handling regulatory, endocrine and paracrine function [2] in addition to its classical lipid storage activity. In the latter role, WAT is considered to be the main repository for fat reserves, in spite of the fact that carefully dissected WAT mass [3] at most justifies about half the body lipid of a normal-weight rat. Muscle contains a sizeable amount of intramyocellular fat [4]; muscle fat also includes significant masses of adipose tissue, infiltrated between and around muscular structures [5]. However, most studies of WAT have been centered on the main WAT macroscopic masses, which are generally assumed to be the main sites of functionality of the adipose organ.

Different WAT sites are known to possess different cell sizes [6], ability to react to hormonal and pharmacological stimuli [7] and proliferative responses [8]. The classical distinction between visceral and subcutaneous or peripheral WAT is closely related to the development/maintenance of the metabolic syndrome [9] and is justified by different metabolic behavior (e.g. the handling of excess lipid) [10]. The implication of immune responsiveness and cytokine secretion have been found to be widely different depending on the WAT site studied [11], which severely limits the actual significance of the conclusions derived from the analysis of data from a single WAT site.

The role of WAT as a regulatory organ, both in the response to challenges to energy homeostasis and the closely-related inflammatory response. have been widely studied [12]. The relevance of non-adipocyte cells in WAT, such as lymphocytes [13], macrophages [14,15], endothelial/vascular or stromatal cells [16] and even nervous terminations [17], may justify in part the different pattern of secretion of apocrine or paracrine signals. The relative presence of non-adipocyte cells is again a factor of site differentiation, since macrophage penetration [18,19], eNOS activity [20] and leptin secretion [21], to cite only a few factors, are site-related. In fact, the protein signals (adipokines and hormones) secreted by WAT are of a widely different nature, and play a large number of different paracrine and endocrine functions [22], affecting the function of vessels, muscle, liver, brain and other organs and systems. This function is closely related to situations of WAT distress, such as obesity [11] and other metabolic syndrome alterations such as insulin resistance and hypertension [23]. WAT response, i.e. secreting short- or medium-term signals, helps modulate (orchestrate?) a concerted response by the whole organism to energy homeostasis challenges.

In the present study we intended to further delve in WAT site-related differences in adipokine signal response induced by a relatively mild (i.e. within the physiological range) metabolic stress: reduced energy availability. The expected responses to a 40% reduction in food intake (a situation comparable to common human dieting schedules but applied to a rodent, which has a higher metabolic rate) should be in line with a reduction of WAT energy content and its emission of energy-preservative signals. As basic model we used male overweight rats [24], which contain sufficient fat to make visible its loss in the short term without the problems posed by the metabolic alterations of a fully settled obesity.

## Methods

### Animals and sample preparation

Adult male Wistar rats were made overweight by a limited period (30 days) of cafeteria diet feeding, as previously described [24]. The rats, initially weighing 350 ± 5 g, were kept under standard conditions of housing and feeding [24].

The animals were kept, handled and killed following the specific procedures approved by the University of Barcelona Animal Welfare and Ethics Committee, in full conformity with the norms and proceedings set forth by the European Union and the Governments of Spain and Catalonia.

Two groups of six rats each were randomly selected: controls and "food-restricted" (FR). The controls had free access to pellet food (maintenance chow, Panlab, Barcelona, Spain). A previous experiment showed that overweight male rats of this size/age ate a mean 18.0 g/d (i.e. 257 kJ/d) of the same rat chow. Since food reduction was set at 40% of the basic diet, FR rats were allowed only 10.8 g per day and rat (i.e. 154 kJ). All rats had water available *ad libitum*. During the experiment, all FR rats ate all the food supplied every day.

On day 10, the rats were killed by decapitation. Blood was allowed to clot, and the serum was stored frozen at -80°C until processed. The following WAT pads were rapidly isolated and completely dissected: intestine-related mesenteric WAT, perigonadal (epididymal), retroperitoneal cordons, and the subcutaneous inguinal fat pads. The samples were blotted and carefully cleaned of extraneous material (epididymis, pancreas, dermis), weighed, frozen in liquid nitrogen and kept at -80°C; this was done in the shortest time possible. The rest of subcutaneous WAT was later dissected and weighed. The dispersion of subcutaneous WAT and the need to excise and process rapidly tissue samples brought us to the compromise of analyzing the well defined inguinal cordons and extend the results to the whole subcutaneous WAT, which complete dissection took more than 25 min per rat. We are aware of this potential source of error, since there are no data available about the metabolic uniformity of different locations of subcutaneous WAT.

Blood serum was used for the measurement of glucose (Trinder kit, Sigma, St. Louis, MO, USA) and non-esterified fatty acids (NEFA kit, Wako Chemicals, Neuss, Germany) by enzymatic-colorimetric methods; total triacylglycerols by a lipase-glycerol kinase spectrophotometric method (kit 11528, Biosystems, Barcelona, Spain); and total cholesterol (both in serum and liver samples) by an enzymatic-colorimetric method (Cholesterol reagent easy, Menarini, Firenze, Italy). Serum samples were also used for the radioimmunoanalysis measurements of leptin (kit RL-83K, Linco, St Charles, MO USA), adiponectin (kit MADP-60HK, Linco), and insulin (kit SRI-13K, Linco).

### Analysis of gene expression

Total WAT tissue RNA was extracted using the Tripure reagent (Roche Applied Science, Indianapolis IN USA), and were quantified in a ND-100 spectrophotometer (Nanodrop Technologies, Wilmington DE USA). Real-time PCR (RT-PCR) amplification was carried out using 0.010 mL amplification mixtures containing Power SYBR Green PCR Master Mix (Applied Biosystems, Foster City, CA USA), equivalent to 8 ng of reverse-transcribed RNA and 300 nM primers. Reactions were run on an ABI PRISM 7900 HT detection system (Applied Biosystems).

Gene expression of perilipin, as well as leptin, adiponectin, interleukin 1β, interleukin 6, TNFα, resistin, visfatin, PAI-1, VEGF-A, MCP1, and the macrophage marker CD68 were estimated using a semiquantitative approach for the ultimate estimation of the number of copies of each expressed gene mRNAs per unit of tissue weight as previously described [25]. In any case, cyclophilin was used as charge control gene in all samples. The list of primers used is given in Table 1.

**Table 1:** Primers used for semiquantitative estimation of gene expression

gene	acronym	forward (5' > 3')	reverse (3' > 5')	bp
cyclophilin A	Ppia	CTGAGCACTGGGGAGAAAGGA	GAAGTCACCACCCTGGCACA	87

perilipin	Plin	GAGGGGCTGATCTGGCTTTG	GCATCTTTTGCCGTCCTGAA	102

leptin	Lep	CGGTTCCTGTGGCTTTGGT	CCGACTGCGTGTGTGAAATG	130

adiponectin	Adipoq	GGAGACGCAGGTGTTCTTGG	AGCCCTACGCTGAATGCTGA	152

resistin	Retn	TCATGCCCAGAACCGAGTTG	CAGCCCCAGGACAAGGAAGA	109

tumor necrosis factor	Tnf	GGCTCCCTCTCATCAGTTCCA	CGCTTGGTGGTTTGCTACGA	104

visfatin	Pbef1	TCTGGAAATCCGCTCGACAC	CACTCCGTCCCCTTGAATGA	129

CD68	Cd68	CCATCCCCACTTGGCTCTCT	TGCGCTGAGAATGTCCACTG	156

MCP1	Cc12	TGCAGGTCTCTGTCACGCTTC	TTCTCCAGCCGACTCATTGG	148

interleukin 1β	Il1b	ATGCCTCGTGCTGTCTGACC	GGCCACAGGGATTTTGTCG	130

interleukin 6	Il6	TGCCTTCTTGGGACTGATGTTG	TGGTCTGTTGTGGGTGGTATCC	97

VEGF-A	Vegfa	CGTCCTGTGTGCCCCTAATG	TGTGCTGGCTTTGGTGAGGT	124

PAI-1	Serpine1	CAGCACACAGCCAACCACAG	GAAGCGAACCCTTTCCCAAA	143

The data were expressed as the absolute amount of the mRNAs corresponding to the given gene in the whole mass of the WAT pad as a way to render comparable the ability to express the gene regardless of cell size (i.e. fat content), but nevertheless considering its physiological significance as an organ. In the case of subcutaneous adipose tissue, the data obtained for the inguinal cordons were applied to the whole tissue mass dissected. Since the four WAT sites selected represent a large majority of the rat's total WAT [3], we assumed that, in the whole, they constituted a fairly representative estimation of that total 'independent' WAT mass, i.e. not associated to other organs (muscle) or structures (vessels).

Statistical comparison between groups and correlations were established by using the unpaired Student's t and linear regression analysis programs of the Prism-5 package (Graphpad Software, La Jolla CA USA). In the linear regression analysis experiments, comparisons of paired parameters were done for each WAT site, including in the analysis all the animals from both dietary groups as one, as a way to determine-using food restriction as metabolic stimulus- the existence of coordinated changes in expressions and metabolite levels.

## Results

**Figure 1:**
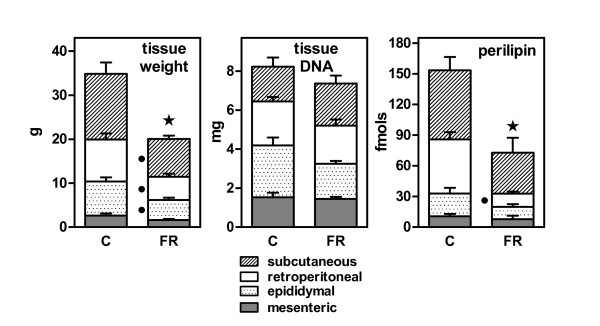
**Effect of 10 days of 40% food restriction on WAT site weight, DNA content, and the expression of perilipin gene**. The data correspond to the mean ± sem of six different animals. Gene expression data are presented as fmols of the corresponding mRNAs in the whole WAT site. C = controls fed *ad libitum*; FR = food restricted. Statistical significance of the differences between data groups. A star indicates an overall (i.e. combined WAT sites) significant (P < 0.05) difference between FR and C. A black dot indicates a significant difference in mass/gene expression for a given WAT site between FR and C.

In the 10-day period studied, controls increased their body weight 2.6 ± 0.4%, whilst the FR rats lost 8.5 ± 0.7%, the differences being statistically significant. Figure 1 shows the weights of WAT sites and the effect of food restriction: total weight of the four WAT sites was reduced by 43% (P < 0.05), but their combined DNA content lost only about 10% (not significant). Figure 1 also shows the sum of perilipin fmols of their corresponding mRNAs in the four WAT sites. Perilipin gene expression decreased in a proportion similar to WAT weight; however, retroperitoneal WAT loss in FR of fat vacuole-lining protein with respect to controls was more marked, in contrast with the more generalized loss of site WAT weight.

Table 2 presents the serum parameters for both groups of rats. Glucose, triacylglycerols, insulin and leptin levels significantly decreased with food restriction, non-esterified fatty acids increased, and there were no changes for cholesterol and adiponectin.

**Table 2:** Serum parameters of overweight male rats subjected to 10 days of reduced food intake.

parameter	units	control	FR
glucose	mM	8.01 ± 0.09	6.02 ± 0.15 *

triacylglycerols	mM	1.58 ± 0.13	0.62 ± 0.05 *

non-esterified fatty acids	mM	0.35 ± 0.03	0.50 ± 0.07 *

total cholesterol	mM	1.36 ± 0.03	1.46 ± 0.11

insulin	pM	574 ± 79	71 ± 10 *

leptin	pM	47.1 ± 1.90	7.1 ± 0.7 *

adiponectin	nM	80.1 ± 5.1	78.1 ± 6.1

The data are the mean ± sem of six different animals. All units are expressed as mmol/L. Differences between groups: * = P < 0.05 versus controls

**Figure 2:**
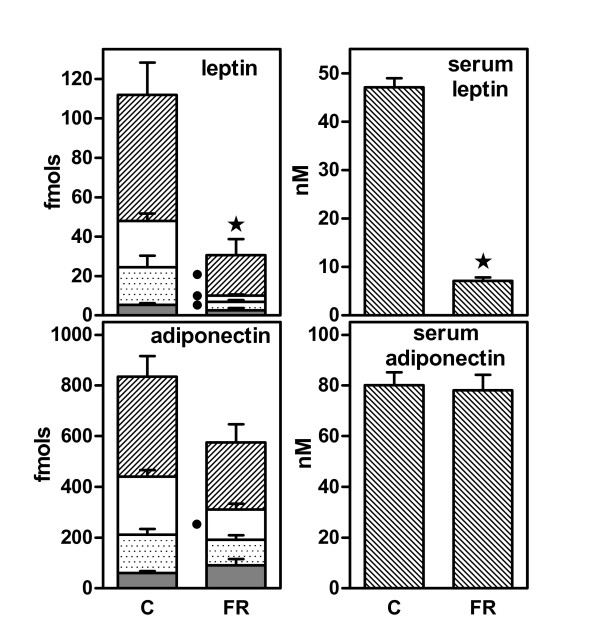
**Effect of 10 days of 40% food restriction on WAT site leptin and adiponectin serum levels and gene expressions**. The data correspond to the mean ± sem of six different animals. The symbols used are the same as in Figure 1. Gene expression data are presented as fmols of the corresponding mRNAs in the whole WAT site. C = controls fed *ad libitum*; FR = food restricted. Statistical significance of the differences between data groups. A star indicates an overall (i.e. combined WAT sites) significant (P < 0.05) difference between FR and C. A black dot indicates a significant difference in mass/gene expression for a given WAT site between FR and C.

In Figure 2, the sum of specific mRNAs for leptin and adiponectin content in the WAT sites are presented in comparison with the serum levels of both adipocytokines. Overall, leptin expression in FR was reduced to a 26% of controls, with serum leptin showing a close 21%, the differences being significant in both cases. The corresponding data for adiponectin were 69% (expression) and 97% (levels), neither being statistically significant. In the case of adiponectin, the only significant decrease in gene expression was observed in retroperitoneal WAT. There was a significant (P = 0.0003) correlation between combined WAT leptin gene expression and the corresponding serum levels when considering all the data (controls and FR) together; this correlation was not significant (P = 0.494) in the case of adiponectin.

**Figure 3:**
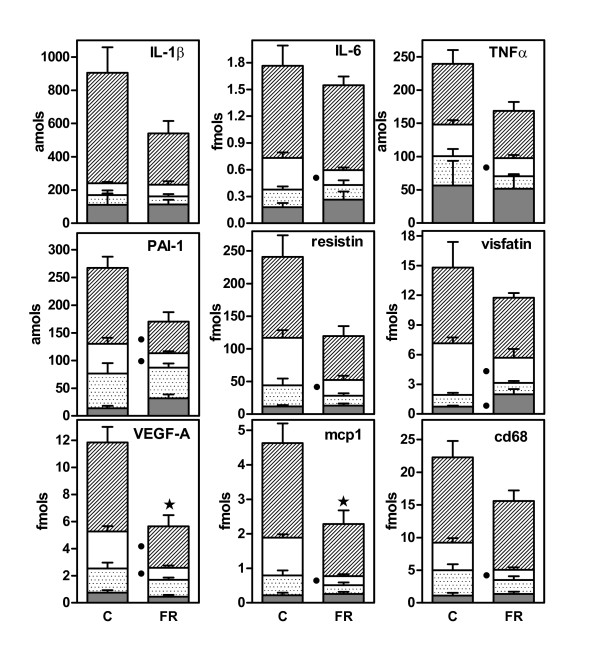
**Effect of 10 days of 40% food restriction on WAT site gene expression of peptide signaling agents**. The data correspond to the mean ± sem of six different animals. The symbols used are the same as in Figure 1. Gene expression data are presented as femtomols or attomols of the corresponding mRNAs in the whole WAT site. C = controls fed *ad libitum*; FR = food restricted. Statistical significance of the differences between data groups. A star indicates an overall (i.e. combined WAT sites) significant (P < 0.05) difference between FR and C. A black dot indicates a significant difference in gene expression for a given WAT site between FR and C.

Figure 3 shows the effect of food restriction on the expression of a number of peptide WAT signals. In all cases, food restriction generated a decrease in the overall specific mRNA content of combined WAT sites that was significant only for VEGF-A and MCP1. There were generalized decreases for all genes tested in retroperitoneal WAT (significant for interleukin 6, TNFα, PAI-1, resistin, visfatin, VEGF-A, MCP1, and CD68). No significant differences were observed for epididymal WAT. Subcutaneous WAT showed a significant decrease in the expression of PAI-1 and VEGF-A genes. Mesenteric WAT presented a significant increase in the expression of the visfatin gene. No significant effects whatsoever were found for interleukin 1β.

Table 3 shows the statistical significance of the correlations between specific mRNA WAT content and either combined tissue weight or tissue DNA. There was a good correlation between cumulative specific mRNA content and WAT weight for all parameters studied, except tissue DNA and visfatin. Individual WAT sites showed different degrees of correlation between gene expression and WAT site weight, with mesenteric WAT being the site with lowest number of correlates. Comparison of specific mRNA content and combined (or site) DNA content resulted in a much lower degree of correlation: the only significant values were found for epididymal WAT, that showed a significant correlation with all parameters except for visfatin and interleukin 6. Mesenteric WAT DNA showed a significant correlation with the specific mRNAs of adiponectin, interleukin 1β, VEGF-A, CD68 and MCP1.

**Table 3:** Statistical significance of the correlations between combined four-site WAT specific mRNA content for peptidic signaling agents and total four-site WAT weight or DNA content (r^2 ^and P values)

adipokine gene expression		total site mRNA copies versus weight					total site mRNA copies versus DNA				
		
		RP	Epi	SC	Me	Total	RP	Epi	SC	Mes	Total
site total DNA	r^2^	0.364	0.929	NS	0.686	**NS**					
									
	P	0.0492	0.0001		0.0016						

perilipin	r^2^	0.594	0.766	0.563	NS	**0.826**	NS	0.824	NS	NS	**NS**
								
	P	0.0091	0.0004	0.0078		**0.0001**		0.0001			

visfatin	r^2^	NS	NS	NS	NS	**NS**	NS	NS	NS	NS	**NS**
											
	P										

leptin	r^2^	0.507	0.745	0.656	0.647	**0.882**	NS	0.809	NS	NS	**NS**
						
	P	0.0139	0.0004	0.0025	0.0028	**0.0000**		0.0002			

resistin	r^2^	NS	0.417	0.579	0.515	**0.468**	NS	0.808	NS	NS	**NS**
								
	P		0.0317	0.0065	0.0013	**0.0203**		0.0004			

adiponectin	r^2^	NS	0.610	0.557	NS	**0.649**	NS	0.706	NS	0.429	**NS**
										
	P		0.0045	0.0083		**0.0027**		0.0012		0.0286	

TNFα	r^2^	0.486	0.628	0.618	NS	**0.523**	NS	0.668	NS	NS	**NS**
								
	P	0.0171	0.0036	0.0041		**0.0119**		0.0021			

interleukin 1β	r^2^	NS	0.386	0.369	NS	**0.861**	NS	0.403	NS	0.415	**NS**
										
	P		0.0413	0.0475		**0.0001**		0.0358		0.0325	

interleukin 6	r^2^	0.487	NS	NS	NS	**0.584**	NS	NS	NS	NS	**NS**
										
	P	0.0168				**0.0062**					

VEGF-A	r^2^	0.523	0.666	0.666	0.609	**0.831**	NS	0.835	NS	0.529	**NS**
						
	P	0.0119	0.0022	0.0021	0.0046	**0.0000**		0.0000		0.0111	

PAI-1	r^2^	0.654	NS	0.805	NS	**0.567**	NS	0.533	NS	NS	**NS**
										
	P	0.0026		0.0004		**0.0075**		0.0107			

CD68	r^2^	0.606	0.511	0.476	0.590	**0.704**	NS	0.499	NS	0.533	**NS**
						
	P	0.0048	0.0134	0.0187	0.0165	**0.0012**		0.0151		0.0107	

MCP1	r^2^	0.488	0.746	0.476	0.455	**0.781**	NS	0.739	NS	0.576	**NS**
						
	P	0.0167	0.0006	0.0187	0.0228	**0.0003**		0.0007		0.0067	

The data are the mean ± sem of six different animals. All units are expressed as mmol/L. Differences between groups: * = P < 0.05 versus controls

Table 4 presents, likewise, the statistical significance of the correlations between combined (i.e. the sum of all WAT sites) specific mRNAs and the serum parameters presented in Table 1. There were no significant correlations for cholesterol, non-esterified fatty acids and adiponectin. However, triacylglycerols, glucose, insulin and leptin were correlated with combined WAT weight, perilipin, leptin, VEGF-A and MCP1. Triacylglycerols were also correlated with the expression of visfatin, adiponectin and CD68. Insulin and leptin were also correlated with resistin, interleukin 1β and PAI-1.

**Table 4:** Statistical significance of the correlations of the serum levels of glucose, lipids, insulin, leptin and adiponectin versus the combined four-site WAT specific mRNA content for peptidic signaling agents and total four-site WAT weight or perilipin expression (r^2 ^and P values)

adipokine gene expression		total site mRNA copies versus serum levels						
		
		cholesterol	NEFA	TAG	glucose	insulin	leptin	adiponectin
total WAT weight	r^2^	NS	NS	0.687	0.442	0.985	0.940	NS
					
	P			0.0030	0.0257	0.0065	0.0016	

perilipin	r^2^	NS	NS	0.391	0.436	0.777	0.835	NS
					
	P			0.0391	0.0259	0.0390	0.0194	

leptin	r^2^	NS	NS	0.543	0.488	0.934	0.969	NS
					
	P			0.0097	0.0167	0.0020	0.0003	

VEGF-A	r^2^	NS	NS	0.497	0.464	0.892	0.931	NS
					
	P			0.0154	0.0210	0.0069	0.0023	

resistin	r^2^	NS	NS	NS	NS	0.885	0.916	NS
							
	P					0.0081	0.0037	

adiponectin	r^2^	NS	NS	0.400	NS	NS	NS	NS
								
	P			0.0491				

visfatin	r^2^	NS	NS	0.419	NS	NS	NS	NS
								
	P			0.0312				

TNFα	r^2^	NS	NS	NS	NS	NS	NS	NS
								
	P							

interleukin 1β	r^2^	NS	NS	NS	NS	0.771	0.834	NS
							
	P					0.0424	0.0197	

interleukin 6	r^2^	NS	NS	NS	NS	NS	NS	NS
								
	P							

PAI-1	r^2^	NS	NS	NS	NS	0.822	0.931	NS
							
	P					0.0233	0.0468	

CD68	r^2^	NS	NS	0.425	NS	NS	NS	NS
								
	P			0.0298				

MCP1	r^2^	NS	NS	0.628	0.389	0.784	0.842	NS
					
	P			0.0036	0.0403	0.0371	0.0175	

The data were calculated taking into account the sum of the four sites for all samples (i.e. from both experimental groups) as a whole. All correlations were positive. TAG = triacylglycerols; NEFA = non-esterified fatty acids. NS = P < 0.05.

## Discussion

The four WAT sites analyzed constitute a large proportion of the total rat's large anatomically distinguishable WAT [3], and its function may be somewhat different from the ancillary role of adipocytes interspersed between other cell types in a number of organs or tissues, such as muscle [5]. WAT role as energy depot, energy availability controller and immune response site [12,22] is commonly attributed to the largest macroscopic adipose tissue groupings that constitute the adipose organ [1].

There is no direct quantitative relationship between gene expression and actual protein synthesis and its eventual secretion, largely because of post-transcriptional regulatory steps, and the control of exocytosis. However, tissue gene expression is often taken as a fairly direct indication of the changes or trends elicited by experimental agents. In our case, the application of a semiquantitarive procedure to evaluate the amount of specific mRNAs [25,26] allowed us to compare tissue sites differing in protein content, total mRNA, cell size and even metabolic activity [27]. Thus, the difficulties of comparing the gene expression data in tissues losing weight due to cell size change and/or apoptosis, can be in part circumvented by estimating the absolute amount of the corresponding mRNAs in whole anatomically well-defined units [27]; in our case four distinct adipose tissue sites.

Mesenteric WAT, probably the only truly visceral WAT, contains the lower proportion of lipids, and its mean cell size is small [27]. Subcutaneous WAT has been found to be a higher leptin secretory site [28] with enhanced leptin expression [29] compared with other WAT depots, a result partly reproduced here; but subcutaneous WAT has also small mean cell size compared with retroperitoneal, but not perigonadal WAT [27].

Restricted feeding affects mainly the WAT lipid (energy) content [24], and to a lower extent WAT cellularity [30]; as a consequence, there is not a good correlation between DNA (i.e. cell) content, perilipin expression (roughly: fat vacuole size maintenance) and lipid (weight). The closer relationship of some of the WAT secreted proteins to one or other of these parameters tend to show whether they are directly affected by energy depletion or simply the changes observed are a consequence of diminished overall WAT cell content.

The only WAT site that lost DNA (albeit not significantly) with food restriction was the epididymal, and thus is the one for which most genes investigated show correlations with DNA content.

Perilipin, a protein related with fat vacuole lining in mature adipocytes [31], was closely correlated with WAT weight, with the striking difference of mesenteric WAT, which function is probably less related to storage of lipids than to dietary lipid processing [27].

Total DNA content of the different WAT sites was fairly similar, but the WAT site weights were not, resulting in much smaller mean cell size for mesenteric WAT than for the other sites [27]. The proportion of specific mRNAs, however did not follow the DNA pattern nor too closely that of weight, in a way that subcutaneous WAT share (38% in controls) accounts for about half of adiponectin, PAI-1, resistin or visfatin (and, perilipin, which suggests a probable relationship between these expressions and vacuole size), or more than half of leptin, interleukins 1β and 6, VEGF-A, MCP1 and CD68 expression.

The comparisons of circulating leptin and adiponectin and tissue gene expression data showed a good direct relationship between quantitative data for gene expression in WAT and circulating leptin levels, a relationship that is maintained in spite of the different effect of food restriction on gene expression (and serum levels). This decrease was directly related to WAT mass, as previously described [32], but the extent of decrease was not uniform for all sites and more marked than the loss of weight. Leptin expression was more correlated with WAT weight (i.e. essentially lipids) than with WAT DNA (i.e. cell numbers), but was also well correlated with circulating energy indicators such as triacylglycerols, glucose and insulin. Most of these relationships have been already described [33], but our results show that probably leptin (levels and expression) is a good index to correlate the level of energy availability, because of its direct relationship with most serum markers.

On the other hand, circulating adiponectin (total) was not correlated with the expression of its regulating gene, probably because active adiponectin largely depends on post-translational molecular weight modification [34], which is not reflected in the analytical procedure followed here. This lack of correlation for adiponectin levels extends to all other parameters studied, with the sole and significant exception of triacylglycerols, in agreement with other studies on the relationships of adiponectin and energy availability [35]. The expression of adiponectin, however, is well correlated with WAT weight (i.e. lipid content), since the correlation was lost when compared with tissue DNA (i.e. cell number) subcutaneous and retroperitoneal, but not for epididymal and mesenteric WAT.

Under basal conditions, visfatin, a tissue-derived proxy for some insulin functions [36], was largely expressed in the retroperitoneal WAT. Visfatin expression was unrelated to weight or DNA, but showed a fair correlation with circulating triacylglycerols (also correlated with WAT weight), which suggests a role for this lipid marker in the control of visfatin.

Interleukin 6 expression was decreased in retroperitoneal WAT, but increased (not significantly) in the mesenteric with food restriction. These changes were unrelated to any of the serum energy indicators studied, marking a difference with interleukin 1β, related to both insulin and leptin. This suggests a more energy-related role for interleukin 1β [37,38] than for interleukin 6, which increase is more related to pathologic situations [39] fairly different from the mild conditions of this restricted feeding. The lack of response of TNFα expression to serum parameters can be interpreted in this same way: a good relationship with tissue weight (except for mesenteric WAT) and no response to serum parameters kept well within the non-pathological range.

Resistin has been considered a marker of insulin resistance [40]; this relationship extends to leptin and WAT weight. However its expression is only related to insulin and leptin levels, not to serum lipid markers. The lack of significant change in its expression under decreased energy availability in most WAT sites hints at resistin not playing a major role in the control of energy availability [41], and only partially to modulate the inflammatory response [42]. The basal conditions tested (dietary overweight) did not give rise to a marked degree of insulin resistance [24,43], and the restriction of food intake used here does not elicit a massive metabolic response that may put in jeopardy the global energy homeostasis of the animal. As a consequence, there was not a full activation of energy stress-related adipokines secretion and/or the immune response to a severe energy deficit challenge. This was reflected in less marked gene expression responses of macrophage attracting factor and interleukins in addition to TNFα.

The case for VEGF-A and PAI-1 is substantially different: they act as angiogenic factors [44] and help control the growth of adipose tissue [45], in spite of having additional functions such as the modulation of the blood coagulation path [46]. VEGF-A is highly correlated to tissue weight and (epididymal and mesenteric WAT) to DNA, but also to glucose, triacylglycerols and the tandem insulin-leptin. This suggests that VEGF-A may be a fair indicator of the energy status of WAT. Its direct relationship with NO synthesis [47] and its function as indicator of endothelial stress [48] point at a probably direct correlation between adipocyte size, growth, and/or energy availability, direct correlated of NO synthesis [49] possibly being mediated by WAT itself by means of VEGF-A.

Mesenteric WAT stands as fairly different from the other sites studied, as previously reported [27], because of its true visceral and mainly processing function. Its smaller mean cell size reflects in part the large share of non-adipocyte cells it contains [12], as a consequence of its role in defense-related functions. The close correlations found for interleukin 1β, the macrophage attracting factor MCP1 [50], and a macrophage marker, CD68 [51] with respect to DNA agree with this interpretation.

The close relationships between a large proportion of the adipokine markers studied under two different situations of WAT energy replenishment with serum parameters: leptin, insulin, triacylglycerols and glucose show that the response of WAT to changing overall energy homeostasis is fairly faster than usually assumed. It also shows that, these serum parameters may be considered as good indicators of the energy status under conditions set within the physiological range.

## Conclusion

The considerable diversity of expression patterns presented suggest that WAT is far from being an uniform energy depot, or even behaving as a single dispersed organ. The main WAT sites studied showed different patterns of regulatory protein gene expression. These data may be considered indicative of their probable patterns of secretion if we take as example the case of leptin, in which we have found a good correlation between serum levels and the combined WAT specific mRNAs content. In addition, we have found that there is a certain degree of WAT site specialization in the differential expression (and probably secretion) of adipokines (Table 5): subcutaneous WAT showed the highest concentration for leptin, CD68 and MCP-1, mesenteric WAT for TNFα (and both tissues for the interleukins 1β and 6); resistin was highly expressed in subcutaneous and retroperitoneal WAT. A relatively mild energy stress, such as a food restriction comparable to human hypocaloric diets, induced different cytokine expression patterns for mesenteric and the other WATs; this may be related to both intestine-derived energy availability and a marked inflammatory-related response. However, retroperitoneal WAT, and to a lower extent subcutaneous and epididymal, reacted decreasing the expression of inflammatory markers and the signaling of decreased energy availability in their stores.

**Table 5:** Patterns of adipokine expression changes induced by food intake restriction in overweight male rats

	mesenteric	subcutaneous	retroperitoneal	epididymal
site of the highest concentration (fmol/g) of the corresponding mRNAs		leptin		
	
		resistin	resistin	
	
	TNFα			
	
		CD68		
	
		MCP-1		
	
	interleukin 1β	interleukin 1β		
	
	interleukin 6	interleukin 6		

statistically significant (P < 0.05) changes induced by food restriction on total tissue content of specific mRNAs		↓ leptin	↓ leptin	↓ leptin
	
			↓ adiponectin	
	
			↓ resistin	
	
			↓ TNFα	
	
	↓ visfatin		↓ visfatin	
	
			↓ CD68	
	
			↓ MCP-1	
	
			↓ interleukin 6	
	
		↓ VEGF-A	↓ VEGF-A	
	
		↓ PAI-1	↓ PAI-1	

The data shown highlight the fact that different WAT sites show different proinflammatory and energy signaling responses to changes in energy availability, and also that WAT sites (or their cells) show too many differences to simply extend to the whole-body WAT the findings obtained on one or even a couple of sites.

This reflection on the considerable diversity of WAT in the expression of regulatory adipokines suggests that the other-smaller, more disperse-WAT sites' role, has to be analyzed and quantified too before we can establish a global role for whole-body WAT.

## Abbreviations used

WAT: white adipose tissue; TNFα: tumor necrosis factor alpha; PAI-1: plasminogen activator inhibitor 1; VEGF-A: vascular endothelial growth factor A; MCP1: monocyte chemnotactic protein 1; CD68: cluster of differentiation 68; NO: nitric oxide.

## Competing interests

The authors declare that they have no competing interests.

## Authors' contributions

The experiment was designed by ME and MA. Animal handling, control and special feeding was carried out by MMR. Sampling was done by all four Authors. Sample preparation, RT-PCR and most of the laboratory analyses were done by MMR, other analyses were done by MMR and ME. Calculations and data processing was done by JAFL, ME and MMR. The analysis of data was done by all four Authors. Initial draft of the manuscript and early conclusions were done by MA. All Authors participated in the final redaction of the manuscript.

## References

[B1] Cinti S The adipose organ Prost Leukot Ess Fatty Acids 2005 73 9 15 10.1016/j.plefa.2005.04.010 15936182

[B2] Badman MK Flier JS The adipocyte as an active participant in energy balance and metabolism Gastroenterol 2007 132 2103 2115 10.1053/j.gastro.2007.03.058 17498506

[B3] Remesar X Fernández-López JA Blay MT Savall P Salas A Díaz-Silva M Esteve M Grasa MM Alemany M Effect of oral oleoyl-estrone on adipose tissue composition in male rats Int J Obesity 2002 26 1092 1102 10.1038/sj.ijo.0802056 12119575

[B4] Schrauwen-Hinderling VB Hesselink MKC Schrauwen P Kooi ME Intramyocellular lipid content in human skeletal muscle Obesity 2006 14 357 367 10.1038/oby.2006.47 16648604

[B5] Gondret F Guitton N Guillerm-Regost C Louveau I Regional differences in porcine adipocytes isolated from skeletal muscle and adipose tissues as identified by a proteomic approach J Anim Sci 2008 86 2115 2125 10.2527/jas.2007-0750 18310487

[B6] Prunet-Marcassus B Cousin B Caton D André M Pénicaud L Casteilla L From heterogeneity to plasticity in adipose tissues: Site-specific differences Exp Cell Res 2006 312 727 736 10.1016/j.yexcr.2005.11.021 16386732

[B7] Shinozaki S Chiba T Kokame K Miyata T Ai M Kawakami A Kaneko E Yoshida M Shimokado K Site-specific effect of estradiol on gene expression in the adipose tissue of ob/ob mice Horm Metabol Res 2007 39 192 196 10.1055/s-2007-970417 17373633

[B8] Tchkonia T Giorgadze N Pirtskhalava T Tchoukalova Y Karagiannides I Forse RA DePonte M Stevenson M Guo W Han JR Waloga G Lash TL Jensen MD Kirkland JL Fat depot origin affects adipogenesis in primary cultured and cloned human preadipocytes Am J Physiol 2002 282 R1286 R1296 10.1152/ajpregu.00653.2001 11959668

[B9] Wajchenberg BL Subcutaneous and visceral adipose tissue: Their relation to the metabolic syndrome Endocr Rev 2000 21 697 738 10.1210/er.21.6.697 11133069

[B10] Votruba SB Jensen MD Regional fat deposition as a factor in FFA metabolism Annu Rev Nutr 2007 27 149 163 10.1146/annurev.nutr.27.061406.093754 17506663

[B11] Maury E Ehala-Aleksejev K Guiot Y Detry R Vandenhooft A Brichard SM Adipokines oversecreted by omental adipose tissue in human obesity Am J Physiol 2007 293 E656 E665 10.1152/ajpendo.00127.2007 17578888

[B12] Alemany M Fernández-López JA Adipose tissue: something more than just adipocytes Curr Nutr Food Sci 2006 2 141 150 10.2174/157340106776818817

[B13] Kintscher U Hartge M Hess K Foryst-Ludwig A Clemenz M Wabitsch M Fischer-Posovszky P Barth TFE Dragun D Skurk T Hauner H Bluher M Unger T Wolf AM Knippschild U Hombach V Marx N T-lymphocyte infiltration in visceral adipose tissue – A primary event in adipose tissue inflammation and the development of obesity-mediated insulin resistance Art Thromb Vasc Biol 2008 28 1304 1310 10.1161/ATVBAHA.108.165100 18420999

[B14] Weisberg SP McCann D Desai M Rosenbaum M Leibel RL Ferrante AW Obesity is associated with macrophage accumulation in adipose tissue J Clin Invest 2003 112 1796 1808 14679176 10.1172/JCI19246 PMC296995

[B15] Ortega Martínez de Victoria E Xu XY Koska J Francisco AM Scalise M Ferrante AW Krakoff J Macrophage content in subcutaneous adipose tissue associations with adiposity, age, inflammatory markers, and whole-body insulin action in healthy Pima lndians Diabetes 2009 58 385 393 10.2337/db08-0536 19008342 PMC2628612

[B16] O'Brien SN Mantzke KA Kilgore MW Price TM Relationship between adipose stromal-vascular cells and adipocytes in human adipose tissue Ann Quant Cytol Histol 1996 18 137 143 8744503

[B17] Bartness TJ Dual innervation of white adipose tissue: some evidence for parasympathetic nervous system involvement J Clin Invest 2002 110 1235 1237 12417560 10.1172/JCI17047 PMC151621

[B18] Curat CA Wegner V Sengenes C Miranville A Tonus C Busse R Bouloumié A Macrophages in human visceral adipose tissue: increased accumulation in obesity and a source of resistin and visfatin Diabetologia 2006 49 744 747 10.1007/s00125-006-0173-z 16496121

[B19] Bourlier V Zakaroff-Girard A Miranville A de Barros S Maumus M Sengenes C Galitzky J Lafontan M Karpe F Frayn KN Bouloumie A Remodeling phenotype of human subcutaneous adipose tissue macrophages Circulation 2008 117 806 815 10.1161/CIRCULATIONAHA.107.724096 18227385

[B20] Rydén M Elizalde M van Harmelen V Öhlund A Hoffstedt J Bringman S Andersson K Increased expression of eNOS protein in omental versus subcutaneous adipose tissue in obese human subjects Int J Obesity 2001 25 811 815 10.1038/sj.ijo.0801625 11439294

[B21] Van Harmelen V Reynisdottir S Eriksson P Thörne A Hoffstedt J Lönnqvist F Arner P Leptin secretion from subcutaneous and visceral adipose tissue in women Diabetes 1998 47 913 917 10.2337/diabetes.47.6.913 9604868

[B22] Breitling R Robust signaling networks of the adipose secretome Tr Endocr Metab 2008 20 1 7 10.1016/j.tem.2008.08.006 18930409

[B23] Antuna-Puente B Feve B Fellahi S Bastard JP Adipokines: The missing link between insulin resistance and obesity Diabet Metab 2008 34 2 11 10.1016/j.diabet.2007.09.004 18093861

[B24] Romero MM Esteve M Alemany M Combined effects of oral oleoyl-estrone and limited food intake on body composition of young overweight male rats Int J Obesity 2006 30 1149 1156 10.1038/sj.ijo.0803224 16418752

[B25] Romero MM Grasa MM Esteve M Fernández-López JA Alemany M Semiquantitative RT-PCR measurement of gene expression in rat tissues including a correction for varying cell size and number Nutr Metab 2007 4 26 10.1186/1743-7075-4-26 PMC2217546 18039356

[B26] Alzón M Mendizabal JA Arana A Alberti P Purroy A Adipocyte cellularity in different adipose depots in bulls of seven Spanish breeds slaughtered at two body weights Animal 2007 1 261 267 10.1017/S1751731107392744 22444292

[B27] Romero MM Fernández-López JA Esteve M Alemany M Site-related white adipose tissue lipid-handling response to oleoyl-estrone treatment in overweight male rats Eur J Nutr 2009 in press (Published online on 27/03/2009, DOI 10.1007/s00394-009-0013-2) 10.1007/s00394-009-0013-2 19326039

[B28] Remesar X Fernández-López JA Blay MT Savall P Salas A Díaz-Silva M Esteve M Grasa MM Alemany M Effect of oral oleoyl-estrone on adipose tissue composition in male rats Int J Obesity 2002 26 1092 1102 10.1038/sj.ijo.0802056 12119575

[B29] Hube F Lietz U Igel M Jensen PB Tornqvist H Joost HG Hauner H Difference in leptin mRNA levels between omental and subcutaneous abdominal adipose tissue from obese humans Horm Metabol Res 1996 28 690 693 10.1055/s-2007-979879 9013743

[B30] Sorisky A Magun R Gagnon AM Adipose cell apoptosis: death in the energy depot Int J Obesity 2000 24 Suppl 4 S3 S7 10.1038/sj.ijo.0801491 11126237

[B31] Brasaemle DL The perilipin family of structural lipid droplet proteins: stabilization of lipid droplets and control of lipolysis J Lipid Res 2007 48 2547 2559 10.1194/jlr.R700014-JLR200 17878492

[B32] Wadden TA Considine RV Foster GD Anderson DA Sarwer DB Caro JS Short- and long-term changes in serum leptin in dieting obese women: Effects of caloric restriction and weight loss J Clin Endocrinol Metab 1998 83 214 218 10.1210/jc.83.1.214 9435444

[B33] Chin-Chance C Polonsky KS Schoeller DA Twenty-four-hour leptin levels respond to cumulative short-term energy imbalance and predict subsequent intake J Clin Endocrinol Metab 2000 86 2685 2691 10.1210/jc.85.8.2685 10946866

[B34] Wang Y Lam KS Chan L Chan KW Lam JB Lam MC Hoo RC Mak WW Cooper GJ Xu AM Post-translational modifications of the four conserved lysine residues within the collagenous domain of adiponectin are required for the formation of its high molecular weight oligomeric complex J Biol Chem 2006 281 16391 16400 10.1074/jbc.M513907200 16621799

[B35] Salmenniemi U Ruotsalainen E Pihlajamäki J Vauhkonen I Kainulainen S Punnonen K Vanninen E Laakso M Multiple abnormalities in glucose and energy metabolism and coordinated changes in levels of adiponectin, cytokines, and adhesion molecules in subjects with metabolic syndrome Circulation 2004 110 3842 3848 10.1161/01.CIR.0000150391.38660.9B 15596567

[B36] Fukuhara A Matsuda M Nishizawa M Segawa K Tanaka M Kishimoto K Matsuki Y Murakami M Ichisaka T Murakami H Watanabe E Takagi T Akiyoshi M Ohtsubo T Kihara S Yamashita S Makishima M Funahashi T Yamanaka S Hiramatsu R Matsuzawa Y Shimomura I Visfatin: A protein secreted by visceral fat that mimics the effects of insulin Science 2005 307 426 430 10.1126/science.1097243 15604363

[B37] Faggioni R Fantuzzi G Fuller J Dinarello CA Feingold KR Grunfeld C IL-1b mediates leptin induction during inflammation Am J Physiol 1998 274 R204 R208 9458919 10.1152/ajpregu.1998.274.1.R204

[B38] Elander L Engstrom L Hallbeck M Blomqvist A IL-1b and LPS induce anorexia by distinct mechanisms differentially dependent on microsomal prostaglandin E synthase-1 Am J Physiol 2007 292 R258 R267 10.1152/ajpregu.00511.2006 16946079

[B39] Wannamethee SG Whincup PH Rumley A Lowe GD Inter-relationships of interleukin-6, cardiovascular risk factors and the metabolic syndrome among older men J Thromb Haemost 2007 5 1637 1643 10.1111/j.1538-7836.2007.02643.x 17596140

[B40] Haluzik M Haluzikova D The role of resistin in obesity-induced insulin resistance Curr Opin Invest Drugs 2006 7 306 311 16625816

[B41] Barb D Wadhwa SG Kratzsch J Gavrila A Chan JL Williams CJ Karchmer AW Mantzoros CS Circulating resistin levels are not associated with fat redistribution, insulin resistance, or metabolic profile in patients with the highly active antiretroviral therapy-induced metabolic syndrome J Clin Endocrinol Metab 2005 90 5324 5328 10.1210/jc.2005-0742 15956078

[B42] Qatanani M Szwergold NR Greaves DR Ahima RS Lazar MA Macrophage-derived human resistin exacerbates adipose tissue inflammation and insulin resistance in mice J Clin Invest 2009 119 531 539 10.1172/JCI37273 PMC2648673 19188682

[B43] Goran MI Lane C Toledo-Corral C Weigensberg MJ Persistence of pre-diabetes in overweight and obese Hispanic children: association with progressive insulin resistance, poor beta-cell function, and increasing visceral fat Diabetes 2008 57 3007 3012 10.2337/db08-0445 18678615 PMC2570397

[B44] Nagy JA Dvorak AM Dvorak HF VEGF-A and the induction of pathological angiogenesis Annu Rev Pathol 2007 2 251 275 10.1146/annurev.pathol.2.010506.134925 18039100

[B45] Lijnen HR Alessi MC van Hoef B Collen D Juhan-Vague I On the role of plasminogen activator inhibitor-1 in adipose tissue development and insulin resistance in mice J Thromb Haemost 2005 3 1174 1179 10.1111/j.1538-7836.2005.01390.x 15946208

[B46] Mertens I Verrijken A Michiels JJ van der Planken M Ruige JB Van Gaal LF Among inflammation and coagulation markers, PAI-1 is a true component of the metabolic syndrome Int J Obesity 2006 30 1308 1314 10.1038/sj.ijo.0803189 16389265

[B47] He H Venema VJ Gy XL Venema RC Marrero MB Caldwell RB Vascular endothelial growth factor signals endothelial cell production of nitric oxide and prostacyclin through Flk-1/KDR activation of c-Src J Biol Chem 1999 274 25130 25135 10.1074/jbc.274.35.25130 10455194

[B48] Charnock-Jones S Vascular endothelian growth factors (VEGFs), their receptors and their inhibition Cell Trans 2005 21 1 6

[B49] Kroll J Waltenberger J A novel function of VEGF receptor-2 (KDR): rapid release of nitric oxide in response to VEGF-A stimulation in endothelial cells Biochem Biophys Res Commun 1999 265 636 639 10.1006/bbrc.1999.1729 10600473

[B50] Kanda H Tateya S Tamori Y Kotani K Hiasa K Kitazawa R Kitazawa S Miyachi H Maeda S Egashira K Kasuga M MCP-1 contributes to macrophage infiltration into adipose tissue, insulin resistance, and hepatic steatosis in obesity J Clin Invest 2006 116 1494 1505 10.1172/JCI26498 16691291 PMC1459069

[B51] Holness CL Simmons DL Molecular cloning of CD68, a human macrophage marker related to lysosomal glycoproteins Blood 1993 81 1607 1613 7680921

